# Multidrug-Resistant Atypical Variants of *Shigella flexneri* in China

**DOI:** 10.3201/eid1907.111221

**Published:** 2013-07

**Authors:** Shaofu Qiu, Yong Wang, Xuebin Xu, Peng Li, Rongzhang Hao, Chaojie Yang, Nan Liu, Zhenjun Li, Zhongqiang Wang, Jian Wang, Zhihao Wu, Wenli Su, Guang Yang, Huiming Jin, Ligui Wang, Yansong Sun, Zhengan Yuan, Liuyu Huang, Hongbin Song

**Affiliations:** Academy of Military Medical Sciences, Beijing, China (S. Qiu, Y. Wang, P. Li, R. Hao, C. Yang, N. Liu, Z. Wang, J. Wang, Z. Wu, W. Su, G. Yang, L. Wang, Y. Sun, L. Huang, H. Song);; Shanghai Municipal Center for Disease Control and Prevention, Shanghai, China (X. Xu, H. Jin, Z. Yuan);; Chinese Center for Disease Control and Prevention, Beijing (Z. Li)

**Keywords:** Shigella flexneri, multidrug resistance, pulse type, sequence type, atypical, variants, China, diarrheal disease, shigellosis, gram-negative bacteria, bacteria, antimicrobial resistance, enteric infections

## Abstract

We identified 3 atypical *Shigella flexneri* varieties in China, including 92 strains with multidrug resistance, distinct pulse types, and a novel sequence type. Atypical varieties were prevalent mainly in developed regions, and 1 variant has become the dominant *Shigella* spp. serotype in China. Improved surveillance will help guide the prevention and control of shigellosis.

Each year worldwide, ≈1.5 million children <5 years of age die from diarrheal diseases (*1*), which is of particular concern in developing countries. *Shigella* spp. are leading bacterial causes of diarrhea, responsible for >80 million cases of bloody diarrhea and 700,000 deaths each year (*2*), and *S. flexneri* serotype 2a has been recognized as the most prevalent serotype in China for many years (*3*). To better understand the etiology of bacterial diarrhea in China and to determine if *S. flexneri* serotype 2a is still the most prevalent serotype in China, we conducted a study during May 2008–December 2010.

## The Study

A total of 10,021 fecal samples were obtained from patients with diarrhea or dysentery at hospitals in 8 provinces within the eastern, southern, western, northern, and central regions of China: Liaoning, Shandong, Jiangsu, Guangdong, Gansu, Sichuan, Xinjiang, and Hebei Provinces. Samples were screened for the presence of *Shigella* spp. by using API 20E strips (bioMérieux, Marcy l’Etoile, France). Serotyping was performed by using 1) an antisera kit specific for all type- and group-factor antigens (Denka Seiken, Tokyo, Japan) and 2) a panel of monoclonal antibodies against *S. flexneri* (MASF; Reagensia AB, Stockholm, Sweden). Antimicrobial susceptibility testing was performed by the disk diffusion method as described (*4*). Genetic relationships were estimated by using multilocus sequence typing, as described (*5*), and pulsed-field gel electrophoresis (PFGE) (*6*).

Overall, 1,109 bacterial pathogens were identified. *Shigella* spp. isolates were the most prevalent, representing 273 (24.6%) of the total. Among the *Shigella* strains, 92 atypical strains, with 3 different serologic agglutination profiles, were identified (Table 1); 1 of these strains was initially identified as the previously described serotype 4c (*7*) because it agglutinated with monovalent anti-IV type antisera and monovalent anti-7,8 group antisera. This serotype was also serologically indistinguishable from *S. flexneri* serotype X variant (SFxv), which was recently reported in China (*8*), because it reacted with MASF B–, MASF 7(8)–, and MASF IV-1 E1037–specific antibodies (Technical Appendix Table 1). However, this serotype could not produce indole, whereas SFxv could. This atypical serotype was provisionally designated *S. flexneri* X variant (−:7,8, E1037), indole-negative variety. We isolated 73 isolates belonging to this atypical variant, which has become the most frequent serotype in China (Table 1). In 2008, the first 19 *S. flexneri* X variant (−:7,8, E1037) isolates were detected in Beijing; then in 2009, 26 were detected in Beijing, and in 2010, 23 were detected in Beijing, 3 in Jiangsu Province, and 2 in Shandong Province.

**Table 1:** Distribution of different *Shigella* spp. in China during 2008–2010 and 1991–2000

Serotype	1991–2000*		May 2008–December 2010
	No. (%) isolates		No. (%) isolates
*S. flexneri*	8,082 (86.2)		208 (76.2)
1a	272 (2.9)		9 (3.3)
1b	325 (3.5)		29 (10.6)
1c	15 (0.2)		0
2a	6,468 (69.0)		64 (23.4)
2b	152 (1.6)		3 (1.1)
3a	255 (2.7)		0
3b	57 (0.6)		0
3c	9 (0.1)		0
4	230 (2.5)		0
5	30 (0.3)		1 (0.4)
6	73 (0.8)		3 (1.1)
x	70 (0.7)		5 (1.8)
y	126 (1.3)		2 (0.7)
X variant (−:7,8, E1037), indole-negative variety	0		73 (26.7)
*S. flexneri* serotype 2 variant (II:3,4,7,8)	0		17 (6.2)
*S. flexneri* untypeable variant (−:E1037)	0		2 (0.7)
*S. sonnei*	1,022 (10.9)		56 (20.5)
*S. dysenteriae*	132 (1.4)		6 (2.2)
*S. boydii*	139 (1.5)		3 (1.1)

*Data are from (*1*).

The second atypical serotype reacted with monovalent anti-IV type antisera but not the group-specific antisera, and it agglutinated with MASF B–specific and MASF IV-1 E1037–specific antibodies. The serotype was provisionally named *S. flexneri* untypeable variant (−:E1037). Only 2 isolates of this variant were identified in 2010: 1 was recovered from Beijing (*9*), and the other was recovered from Jiangsu Province.

The third atypical serotype was provisionally designated *S. flexneri* serotype 2 variant (II:3,4,7,8) because it could agglutinate with monovalent anti-II type antisera and monovalent anti-3,4 and anti-7,8 group antisera. It also reacted with type-specific antibody MASF II and group-specific antibodies MASF Y-5 and MASF 7,8 (Technical Appendix Table 1). A total of, 17 isolates of serotype 2 variant (II:3,4,7,8) have been detected in Jiangsu Province: 10 in 2008, 4 in 2009, and 3 in 2010.

The atypical serotype strains were all multidrug resistant to ampicillin, ampicillin/sulbactam, chloramphenicol, nalidixic acid, and tetracycline; a total of 91.3% were also resistant to amoxicillin/clavulanic acid, and 84.8% were resistant to trimethoprim/sulfamethoxazole (Table 2; Technical Appendix Table 2). The atypical strains also showed reduced susceptibility to fluoroquinolones and third-generation cephalosporins. Of the X variant (−:7,8, E1037), indole-negative variety strains, 82.2% were resistant to norfloxacin, 39.7% to ciprofloxacin, 21.9% to levofloxacin, and 13.7% to cefotaxime. Of the serotype 2 variant (II:3,4,7,8) strains, 29.4% were resistant to norfloxacin, 29.4% to ciprofloxacin, 17.6% to levofloxacin, and 17.6% to cefotaxime. The 2 *S. flexneri* untypeable variant (−:E1037) strains were also resistant to norfloxacin and ciprofloxacin. Furthermore, 6 of the X variant (−:7,8, E1037), indole-negative variety strains were also resistant to ciprofloxacin and cefotaxime.

**Table 2:** Antimicrobial drug resistance profiles of variant serotypes of *Shigella flexneri* isolates recovered from patients with diarrhea, China, May 2008–December 2010*

Antimicrobial drug	% Resistant isolates														
	X variant (−:7,8, E1037), indole-negative variety, n = 73						Serotype 2 variant (II:3,4,7,8), n = 17						Untypeable variant (−:E1037), n = 2		
	R	I			S		R		I		S		R	I	S
Ampicillin	100.0	0			0		100.0		0		0		100.0	0	0
Ampicillin/sulbactam	100.0	0			0		100.0		0		0		100.0	0	0
Chloramphenicol	100.0	0			0		100.0		0		0		100.0	0	0
Nalidixic acid	100.0	0			0		100.0		0		0		100.0	0	0
Tetracycline	100.0	0			0		100.0		0		0		100.0	0	0
Amoxicillin/clavulanic acid	91.8	5.5			2.7		88.2		0		11.8		100.0	0	0
Trimethoprim/sulfa	90.4	0			9.6		58.8		0		41.2		100.0	0	0
Norfloxacin	82.2	16.4			1.4		29.4		0		70.6		100.0	0	0
Ciprofloxacin	39.7	49.3			11.0		29.4		0		70.6		100.0	0	0
Levofloxacin	21.9	26.0			52.1		17.6		11.8		70.6		0	50.0	50.0
Cefotaxime	13.7	13.7			72.6		17.6		5.9		76.5		0	0	100.0
Gentamicin	6.8	0			93.2		5.9		0		94.1		0	0	100.0
Ceftazidime	5.5	2.7			91.8		0		0		100.0		0	0	100.0
Imipenem	2.7		0	97.3			0	0		100.0			0	0	100.0

*R, resistant; I, intermediate; S, susceptible.

Multilocus sequence typing identified all of the variant strains as a new sequence type (ST), ST100, which differs from ST18 at the *lysP* locus only (Technical Appendix Figure). Other serotypes (e.g., 1a, 2a, 2b, and Y) were also identified as ST100. The 73 isolates of X variant (−:7,8, E1037), indole-negative variety were typed into 29 distinct pulse types (PTs) by PFGE analysis, and the 2 *S. flexneri* untypeable variant (−:E1037) isolates belonged to different PTs and had relatively low similarity (Technical Appendix Figure). The 17 serotype 2 variant (II:3,4,7,8) isolates grouped into 17 PTs, showing high genetic diversity. Several serotype 2 variant (II:3,4,7,8) isolates had greater genetic distance from other serotype 2 isolates. In particular, PT70 had a genetic similarity of <65% with other serotype 2 variant (II:3,4,7,8) isolates.

## Conclusions

Three varieties of *S. flexneri* serotypes, including 92 atypical strains, were identified in this study. The X variant (−:7,8, E1037), indole-negative variety displayed a serologic agglutination profile indistinguishable from that for SFxv, but the indole-negative variety had different biochemical reactions, more serious drug resistance, and different ST and PTs. All variant strains were identified as ST100, a new ST containing multiple other serotypes (e.g., 1a, 2a, 2b, and Y). This finding demonstrates that ST100 is the predominant ST circulating among different *S. flexneri* serotypes in China. Moreover, the 3 atypical varieties were also genetically distinct by PFGE analysis and showed a relatively high level of genetic diversity; thus, these *S. flexneri* varieties may have existed for a long time or experienced frequent genetic mutations.

The X variant (−:7,8, E1037), indole-negative variety serotype has supplanted serotype 2a and *S. sonnei* (*3*) and represents a new dominant *S. flexneri* serotype in China; serotype 2 variant (II:3,4,7,8) has become the fifth most common serotype of *Shigella* spp. in China. The variant strains were prevalent mainly in Beijing and in Jiangsu and Shandong Provinces; these provinces, located in the eastern and northern regions of China, have more developed economies and larger populations compared with the provinces in western China. Industrialization, trade, frequent movement of population, and environmental change can cause the shifting prevalence of diarrheal pathogens and lead to the dissemination of foodborne diseases worldwide (*10*,*11*). Therefore, the dynamic change in *S. flexneri*, especially the emergence of new serotypes, may be attributable to the economy and trade development or to human migration.

Of particular concern is that the atypical strains were completely resistant to several antimicrobial drugs used to treat shigellosis in China (*12*), and they showed reduced susceptibility to fluoroquinolones and third-generation cephalosporins. The World Health Organization has recommended ciprofloxacin as a first-line antimicrobial drug for shigellosis treatment (*12*), and third-generation cephalosporins are considered as alternative drug treatments (*13*). However, the *S. flexneri* resistance to ciprofloxacin and cefotaxime that we detected in this study was higher than previously reported in China (*8*,*14*); this finding raises serious questions regarding the effective treatment of shigellosis in the future.

In conclusion, 3 atypical *S. flexneri* serotypes with extensive multidrug resistance, distinct PTs, and a novel ST were identified in China. Continuous surveillance should be encouraged to determine the changing trends of these variants in geographic, temporal, phenotypic, and genotypic patterns. Such knowledge will improve our understanding of the actual level of disease and provide guidance for the prevention and control of shigellosis.

Technical Appendix Agglutination reactions of *Shigella flexneri* variant serotypes and reference strains collected in China during May 2008–December 2010, antimicrobial drug resistance of atypical *Shigella* spp. collected in China during 1991–2000 and 2008–2010, and pulsed-field gel electrophoresis dendrogram of *S. flexneri* subtypes.
