# Supplementary material for: Multidrug-Resistant Atypical Variants of Shigella flexneri in China
Source: Emerg Infect Dis. 2013 Jul;19(7):1147–50. doi: 10.3201/eid1907.111221 (PMC3713959; doi:10.3201/eid1907.111221)
Supplement: Technical Appendix — Agglutination reactions of Shigella flexneri variant serotypes and reference strains collected in China during May 2008–December 2010, antimicrobial drug resistance of atypical Shigella spp. collected in China during 1991–2000 and 2008–2010, and pulsed-field gel electrophoresis dendrogram of S. flexneri subtypes. [file 11-1221-Techapp-s1.pdf]

Article DOI: http://dx.doi.org/10.3201/eid1907.111221

Multidrug-Resistant Atypical Variants of *Shigella flexneri* in China

Technical Appendix

Technical Appendix Table 1. Agglutination reactions of the variant serotypes of *Shigella flexneri* and reference strains tested during a study of multidrug-resistant atypical variants of *Shigella flexneri* in China. May 2008–December 2010 *

		Antisera specific for all type- and group-factor antigens†									Panel of monoclonal antibodies against *S. flexneri*‡										
		Typing sera						Grouping sera			Type antigen specific					Group antigen specific					
Isolate	Serotype	I	II	III	IV	V	VI	(3)4	6	7(8)	I	II	IV:2	V	VI	Y-5	6	7,8	B	IV:1	1c
Sf301	2a	–	+	–	–	–	–	+	–	–	–	+	–	–	–	+	–	–	+	–	–
ATCC 4a	4a	–	–	–	+	–	–	+	–	–	–	–	+	–	–	+	-	–	+	+	–
NCTC 4b	4b	–	–	–	+	–	–	–	+	–	–	–	+	–	–	–	+	–	+	–	–
Shig0001	SFxv	–	–	–	+	–	–	–	–	+	–	–	–	–	–	–	–	–	+	+	–
Shig0004	*S. flexneri* untypeable variant (–:E1037)	–	–	–	+	–	–	–	–	–	–	–	–	–	–	–	–	–	+	+	–
Shig0083	*S. flexneri* untypeable variant (–:E1037)	–	–	–	+	–	–	–	–	–	–	–	–	–	–	–	–	-	+	+	–
Shig0008	X variant (–:7,8, E1037), indole-negative variety	–	–	–	+	–	–	–	–	+	–	–	–	–	–	–	–	+	+	+	–
Shig0009	X variant (–:7,8, E1037), indole-negative variety	–	–	–	+	–	–	–	–	+	–	–	–	–	–	–	–	+	+	+	–
Shig0190	*S. flexneri* serotype 2 variant (II:3,4,7,8)	–	+	–	–	–	–	+	–	+	–	+	–	–	–	+	–	+	+	–	–
Shig0191	*S. flexneri* serotype 2 variant (II:3,4,7,8)	–	+	–	–	–	–	+	–	+	–	+	–	–	–	+	–	+	+	–	–

*SFxv, *S. flexneri* serotype X variant.
†From Denka Seiken, Tokyo, Japan.
‡MASF (Reagensia AB, Stockholm, Sweden).

Technical Appendix Table 2. Antimicrobial drug resistance of atypical *Shigella* spp. collected during 1991–2000 and 2008–2010, China

Antibiotic	% Resistant *Shigella* spp. isolates collected during 1991–2000*	% Resistant *S. flexneri* variants collected during May 2008–December 2010†				
		S. flexneri X variant (–:7,8, E1037), indole-negative variety (n = 73)	*S. flexneri* serotype 2 (II:3,4,7,8) (n =17)	Untypeable *S. flexneri* (–:E1037) (n = 2)	Other *S. flexneri* (n = 78)	*S. sonnei* (n = 56)
Ampicillin	53.0	100.0	100.0	100.0	96.2	92.9
Ampicillin/sulbactam	–	100.0	100.0	100.0	73.1	62.5
Chloramphenicol	18.0	100.0	100.0	100.0	93.6	10.7
Nalidixic acid	–	100.0	100.0	100.0	100.0	100.0
Tetracycline	–	100.0	100.0	100.0	94.9	85.7
Amoxicillin/clavulanic acid	–	91.8	88.2	100.0	69.2	58.9
Trimethoprim/sulfa	62.0	90.4	58.8	100.0	75.6	89.3
Norfloxacin	4.9.0	82.2	29.4	100.0	32.1	8.9
Ciprofloxacin	20.0	39.7	29.4	100.0	23.1	5.4
Levofloxacin	–	21.9	17.6	0	15.4	1.8
Cefotaxime	–	13.7	17.6	0	17.9	25.0
Ceftazidime	–	5.5	0	0	5.1	8.9
Gentamicin	13.0	6.8	5.9	0	14.1	85.7
Imipenem	–	2.7	0	0	0	0

*From (*1*).
†Variants were collected from patients with diarrhea. –, not detected.

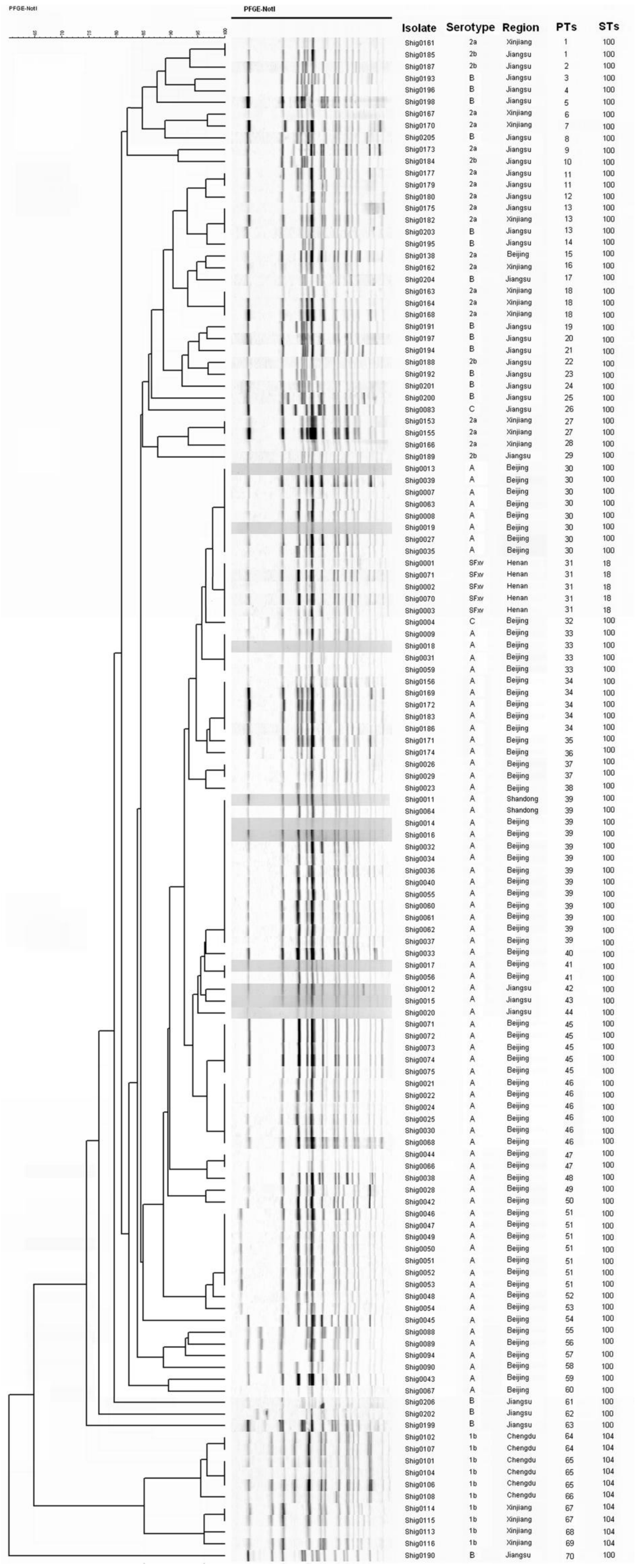

Technical Appendix Figure. Pulsed-field gel electrophoresis dendrogram of *Shigella flexneri* subtypes. The genetic relatedness of atypical and previously known serotypes of *S. flexneri* is shown and was determined by using the unweighted pair group method with arithmetic mean and pairwise Dice coefficients. The strain number, serotype, region, pulse types (PTs), and strain types (STs) are shown for each strain. Serotypes A, B, and C represent the X variant (–:7,8, E1037), indole-negative variety; serotype 2 variant (II:3,4,7,8); and *S. flexneri* untypeable variant (–:E1037), respectively.

Reference

1. Wang XY, Tao F, Xiao D, Lee H, Deen J, Gong J, et al. Trend and disease burden of bacillary dysentery in China (1991–2000). Bull World Health Organ. 2006;84:561–8. PubMed http://dx.doi.org/10.2471/BLT.05.023853
